# Luminal enhancement in intracranial aneurysms: fact or feature?—A quantitative multimodal flow analysis

**DOI:** 10.1007/s11548-021-02486-y

**Published:** 2021-09-14

**Authors:** Franziska Gaidzik, Mariya Pravdivtseva, Naomi Larsen, Olav Jansen, Jan-Bernd Hövener, Philipp Berg

**Affiliations:** 1 Laboratory of Fluid Dynamics and Technical Flows, Otto-von-Guericke University, University of Magdeburg, Forschungscampus STIMULATE, Universitätsplatz 3, 39106 Magdeburg, Germany; 2 Section Biomedical Imaging, Molecular Imaging North Competence Center (MOIN CC), Kiel University, Kiel, Germany; 3 Department of Radiology and Neuroradiology, University Medical Center Schleswig-Holstein (UKSH), Kiel, Germany

**Keywords:** Intracranial aneurysms, Luminal enhancement, VW-MRI, Computational fluid dynamics, Phase-contrast-MRI

## Abstract

**Purpose:**

Intracranial aneurysm (IA) wall enhancement on post-contrast vessel wall magnetic resonance imaging (VW-MRI) is assumed to be a biomarker for vessel wall inflammation and aneurysm instability. However, the exact factors contributing to enhancement are not yet clarified. This study investigates the relationship between luminal enhancement and intra-aneurysmal flow behaviour to assess the suitability of VW-MRI as a surrogate method for determining quantitative and qualitative flow behaviour in the aneurysm sac.

**Methods:**

VW-MRI signal is measured in the lumen of three patient-specific IA flow models and compared with the intra-aneurysmal flow fields obtained using phase-contrast magnetic resonance imaging (PC-MRI) and computational fluid dynamics (CFD). The IA flow models were supplied with two different time-varying flow regimes.

**Results:**

Overall, the velocity fields acquired using PC-MRI or CFD were in good agreement with the VW-MRI enhancement patterns. Generally, the regions with slow-flowing blood show higher VW-MRI signal intensities, whereas high flow leads to a suppression of the signal. For all aneurysm models, a signal value above three was associated with velocity values below three cm/s.

**Conclusion:**

Regions with lower enhancements have been correlated with the slow and high flow at the same time. Thus, further factors like flow complexity and stability can contribute to flow suppression in addition to the flow magnitude. Nevertheless, VW-MRI can qualitatively assess intra-aneurysmal flow phenomena and estimate the velocity range present in the corresponding region.

## Introduction

The prevalence of intracranial aneurysms (IA) in the adult population is estimated to be around 3% [1, 2]. Although IAs are generally associated with a relatively low risk of rupture, haemorrhage following the rupture of aneurysms is fatal in most cases. Therefore, incidental unruptured aneurysm IAs cause significant concerns [3]. Simultaneously, the treatment options available are associated with a variety of severe complications. Thus, a patient-specific IA rupture risk assessment is inevitable.

Previous studies have identified numerous factors that are associated and correlated with a high risk of rupture such as morphological parameters (e.g., size and shape) [4, 5] and flow characteristics [6].

Intracranial blood flow covers a large variety of different flow conditions (slow, stagnant or transitional [7, 8]). Especially, low flow conditions near the aneurysm’s wall have been associated with rupture sites [9]. Moreover, concentrated inflow jets and small impingement regions have been found to instabilize aneurysms [10]. Other flow-related parameters, such as the low shear area (LSA), the oscillatory shear index (OSI) and the neck inflow rate (NIR), contribute to the growth and rupture risk of the aneurysm [9, 11, 12].

Wall inflammation is a primary factor contributing to instable vessel wall constitution and hence instability of aneurysm and change of aneurysm shapes [13–17], but until now, wall inflammation is difficult to assess. However, several studies have suggested using wall enhancement in contrast-enhanced vessel wall magnetic resonance imaging (VW-MRI) as an indirect marker for wall inflammation [18–20]. Exemplarily, different flow conditions (e.g., at the neck, aneurysm body or dome) were associated with a varying degree of local enhancement [21]. Furthermore, VW-MRI in combination with flow MRI was used to correlate morphology and haemodynamics in IAs [22].

Although it is well known that VW-MRI is based on suppressing intraluminal signal, generating images with black blood and bright surrounding tissues, the suppression depends on local flow conditions and works best for higher flow regions. Therefore, inflammation and slow flow can contribute to enhanced luminal regions on VW-MRI [23].

Recent studies have discussed the interaction of slow-flowing blood and enhanced wall regions [3, 24–26]. The slow flow at inner boundaries could lead to an overestimation of the aneurysm wall enhancement. Alternatively, the signal originating from unsuppressed blood may be mistaken for the aneurysm wall [24, 25]. This complicates the distinction between the effect and extent of slow flow and wall inflammation on the enhancement in *in vivo* studies. However, as discussed above, slow flow itself might be a valuable marker for aneurysm instability. Flow in aneurysms can be measured non-invasively using phase-contrast magnetic resonance imaging (PC-MRI). However, the widespread of this technique in clinical facilities is limited by required long examination times. Therefore, we hypothesize that the ability to visualize slow flow by VW-MRI can be a useful feature rather than disadvantage.

Recently, the effect of slow-flowing blood on VW-MRI data alone was investigated *in vitro* using phantom models [24, 27]. However, the exact interaction of flow constitution and blood signal intensity and a possible quantitative correlation remains unclear. Therefore, this study investigates the relationship between flow characteristics and luminal enhancement in VW-MRI measurements by performing an advanced flow analysis. We aim to assess the suitability of VW-MRI as a surrogate method for determining quantitative and qualitative flow behaviour by correlating VW-MRI signal void and flow constitution. The focus is set on the possibility to extract features from VW-MRI that complement or even substitutes information from PC-MRI or numerical flow data. PC-MRI and VW-MRI images have been acquired in phantom models of three patient-specific intracranial aneurysms under varying inflow conditions. Detailed haemodynamic flow simulations based on computational fluid dynamics (CFD) of the respected patient models complement the data and allow for a more detailed analysis.

## Materials and methods

### Case selection

Since IAs show a large variety with respect to size and shape, the intraluminal flow patterns can drastically differ (e.g., stable versus complex haemodynamics). Therefore, three representative IA patients with different sizes and locations as well as *in vivo* VW-MRI enhancement were identified. Due to these differences a large range of flow regimes could be covered and investigated both experimentally and numerically. This enables a more generalized identification of the correlation between signal intensity and velocity values. The patients underwent 3D rotational angiography (3D RA, Allura XperFD 20/10, Philips, Best, the Netherlands) with a spatial resolution of 0.27 mm^3^ as part of the clinical care.

**Fig. 1:**
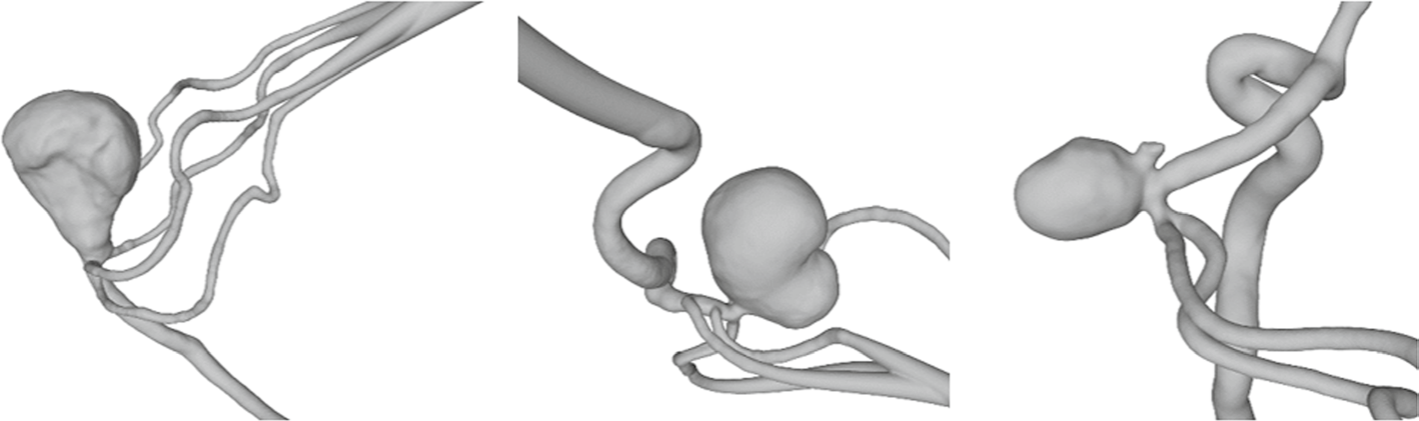
M1—basilar tip aneurysm (left); M2—carotid terminus aneurysm (middle); M3—middle cerebral artery bifurcation aneurysm (right)

Afterwards, three aneurysm flow models (Table 1, Fig. 1) were designed and produced in-house from rigid material (Form 3, Formlabs, USA). First, the vessel lumen was segmented from patient 3D RA. Second, it was simplified, e.g., by cutting branches smaller than 1 mm. Finally, the outer wall (3 mm thick) was added and models were equipped with flow connectors [28]. For further details the interested reader is referred to [27].

**Table 1:** Location, size and patient information of the three aneurysm models used for the underlying study. Note that the case selection is based on prominent locations regarding IA occurrence

Model	Location	Size	Age, Gender
M1	Basilar tip	(38 × 28 mm^2^)	58 years, female
M2	Carotid terminus	(36 × 25 mm^2^)	57 years, female
M3	Middle cerebral artery bifurcation	(12 × 10 mm^2^)	65 years, male

### MRI in vitro measurement set-up

A glycerol–water mixture (40:60) with 0.3 mmol/l of contrast agent (Gadovist, Bayer Vital, Germany) was pumped (Ismaltec MCP Standart, Cole Parmer, USA) through the models. The pump generated two different pulsating inflow profiles (high and low flow conditions) with an averaged flow rate of 160 and 266 ml/min for M1; 203 and 307 ml/min for M2; 208 and 311 ml/min for M3, respectively. Time-dependent flow and pressure were measured at the outlets of the models using a clamp-on transonic flow sensor (ME8PXL-M12, Transonic System Inc., USA) and a Luer-lock pressure sensor (PRESS-N-000, PendoTech, USA).

The *in vitro* MRI measurements were taken on a 3 T MR system (Ingenia CX, R5 V6.1, Philips Healthcare, Best, the Netherlands). The MR protocol comprises VW-MRI and PC-MRI (Table 1). PC-MRI acquired time-resolved three-dimensional and three-directional velocity distribution using a four-point phase-contrast encoding scheme [29].

Further details regarding the measurements and the underlying concept of black blood MRI can be found in [27].

### Haemodynamic flow simulations

High-resolution image-based blood flow simulations were carried out to support the given PC-MRI data in information and resolution. The inner vascular lumens segmented from the patient's 3D RA were used to extract the simulation geometries. The extrusion length of in- and outlet cross sections ranged between 7 and 24 cm to match the distance between the aneurysm model and the flow or pressure sensors, respectively, providing realistic boundary conditions for the CFD simulations. The numerical meshes consisted of polyhedral and prismatic cells and had a base size of 0.1 mm. Spatial discretization was carried out using the finite volume solver STAR-CCM + 14.04 (Siemens Product Lifecycle Management Software Inc., Plato, TX, USA) [30]. The base size was enlarged for the extruded parts of the geometry to reduce the total number of required cells. In total, the resulting meshes consisted of approximately 9.8 million (model M1), 15.1 million (model M2) and 8.5 million (model M3) cells, respectively, guaranteeing a mesh-independent solution. The time-dependent flow rates measured using the transonic flow sensors were applied to the corresponding inlet cross-sections. In addition, the curves provided by the pressure sensors defined the outlet boundary conditions. All vessel walls were assumed to be rigid and no-slip boundary conditions were applied. Fluid properties were considered incompressible (ρ = 1114.5 kg/m^3^), and Newtonian (η = 3.72 mPas) and laminar flow conditions were assumed, which matches the experimental settings. Each of the six time-dependent blood flow simulations comprises three cardiac cycles (time step size Δt = 0.001 s) to obtain a periodic solution and only the last one was included in the analysis. Consistent with the PC-MRI measurements, 24 evenly distributed time points throughout the cardiac cycle were exported and averaged (Table 2).

**Table 2:** MRI protocols used in the current study

	VW-MRI *in vitro*	PC-MRI *in vitro*
Sequence type	3D T1w	3D T1w PC GRE
	Variable flip angle VISTA	
FOV [mm^3^]	120 × 120 × 70	120 × 120 × 70
Acquired voxel [mm^3^]	0.7 × 0.7 × 0.7	1 × 1 × 1
TE/TR [ms]	28/700	4.6/7.5
Number of cardiac phases [ms]	*N/A*	24
V_enc_ [cm/s]	*N/A*	M1: 50 and 100
		M2: 50
		M3: 50

T1w—T1-weighted; VISTA—Volume Isotropic Turbo spin echo Acquisition; PC GRE—Phase-Contrast Gradient echo; FOV—field of view, TE—echo time; TR—repetition time; ${\text{V}}_{{{\text{enc}}}}$—velocity encoding.

### Data processing and flow evaluation

To ensure quantitative comparison between the three modalities (VW-MRI, PC-MRI and CFD), several processing steps are needed. First, velocity, which is encoded in the raw phase difference PC-MRI data, was calculated (GTflow, Version 3.1.12, Gyrotools, Switzerland), considering eddy-current correction and phase-aliasing [31]. Second, the VWI signal intensity values of the vessel lumen data were normalized by the signal intensity of the stationary tissue. Third, the CFD results were manually co-registered with both imaging datasets for all three patient-specific aneurysm models. Finally, each aneurysm sac was virtually separated from the surrounding vessels. The mentioned processing steps as well as the qualitative evaluation were carried out in EnSight v10.2 (ANSYS Inc., Canonsburg, PA, USA).

The subsequent quantitative evaluation was performed in MATLAB R2020a (MathWork, USA) and was based on the voxel resolution of the PC-MRI data. Therefore, VW-MRI and CFD data were downsampled to 1 mm^3^. Furthermore, all voxels closer than 1 mm to the vessel wall, which is the acquired voxel size of PC-MRI data, were discarded in the quantitative analyses. Hence, the influence of partial volume effects near the vessel wall was minimized.

#### Cluster generation

To assess the relationship between signal intensity in the VW images and the measured velocity in each case, the signal values were clustered into ten groups. First, the VW-MRI signal values were combined into ten equidistant regions from the lowest to the highest measured signal value. Second, for each VW-MRI cluster the corresponding PC-MRI values were assigned. Finally, the average signal intensity and velocity calculated for each cluster were compared (Fig. 5). Note that the number of signal values is not equally distributed over the whole range (e.g., mostly low signal values exist in model M1). Therefore, the number of values belonging to one cluster differs within one model (see also Fig. 6).

#### Assessment of velocity fluctuations

The oscillatory velocity index (OVI) can quantify flow pattern variations and flow complexity during the cardiac cycle (OVI ≈ 0 when the velocity field direction remains steady during the cardiac cycle, OVI ≈ 0.5 for high temporal changes and strongly varying flow patterns) [32–34]

1 $$ OVI = \frac{1}{2}\left( {1 - \frac{{\left| {\mathop \smallint \nolimits_{0}^{T} fv_{i} dt} \right|}}{{\mathop \smallint \nolimits_{0}^{t} \left| {fv_{i} } \right|dt}}} \right) $$

where *fv*_*i*_ is the velocity vector comprising of the components and *T* is the duration of one cardiac cycle.

## Results

The consideration of time- and magnitude-varying inflow profiles, as well as the use of differently sized and shaped intracranial aneurysms, located at the different arteries, ensures the coverage of a broad range of flow regimes in this study.

### Luminal enhancement

**Fig. 2:**
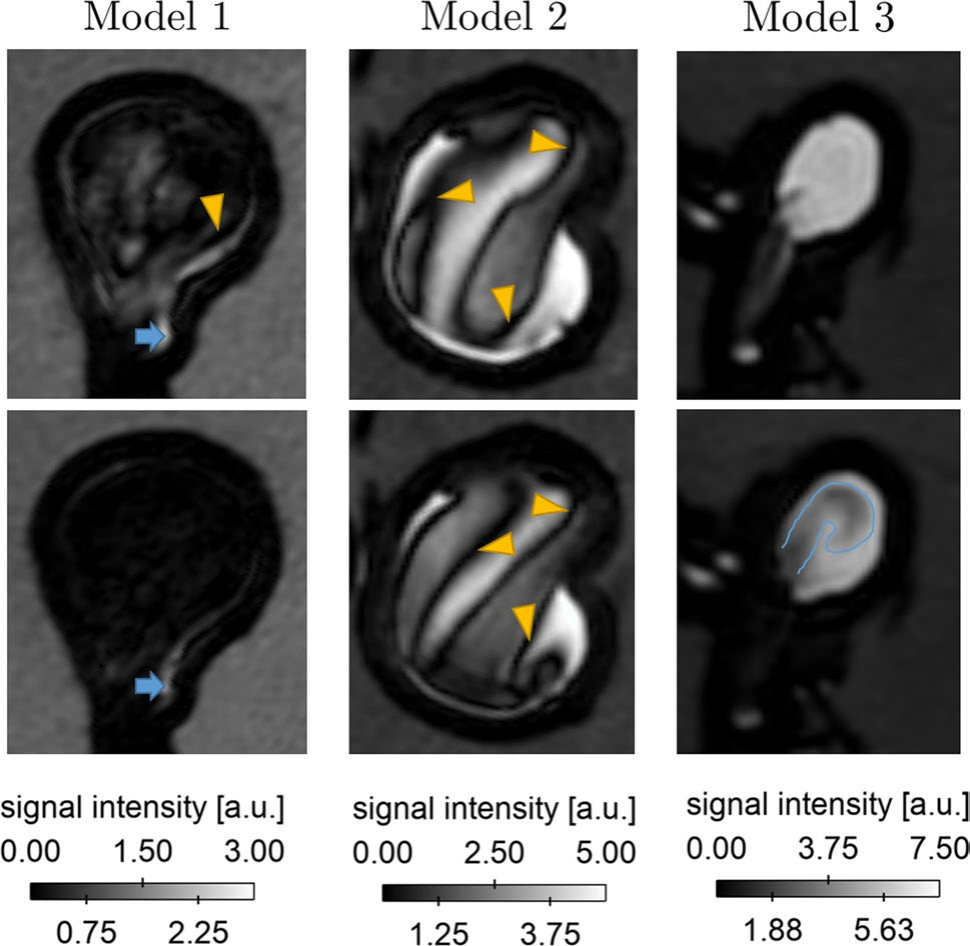
Representative VW-MR images illustrating the enhancement and flow suppression in the aneurysm lumens for all models. Top: Measurements acquired in low flow configuration. Bottom: Measurements acquired in high flow configuration. Signal void zones are marked with yellow triangles, enhanced regions are represented in blue arrows or regions

VW-MRI reveals different levels and patterns of luminal enhancement among the models, which is qualitatively illustrated in Fig. 2. Model M1 (basilar tip aneurysm) experiences the highest suppression over the entire aneurysmal lumen. During the high flow condition, only a small non-suppressed zone near the aneurysm neck remains. Slightly higher signal intensities appear for the lower flow conditions in the same model. Here, inhomogeneous luminal enhancement appears in the centre of the aneurysm. Model M2 (internal carotid artery aneurysm) presents complex enhancement and suppression patterns. Signal void zones separate weak and strong enhanced regions for both flow conditions. Model M3 (MCA bifurcation aneurysm) possesses the highest enhancement. Nearly the complete aneurysm is enhanced during low flow conditions. With the higher flow rate, a regional suppression is observed near the aneurysm neck that appears to be formed like an inflow jet. The luminal enhancement's general characteristics were similar within the model supplied with the different flows, but changed drastically among different models. This can be attributed to the difference in size and shape. Moreover, the actual flow rate entering the aneurysm is highly different due to the presence of the side branches at models 2 and 3 and the structure of the parent vessels.

### Flow characteristics

**Fig. 3:**
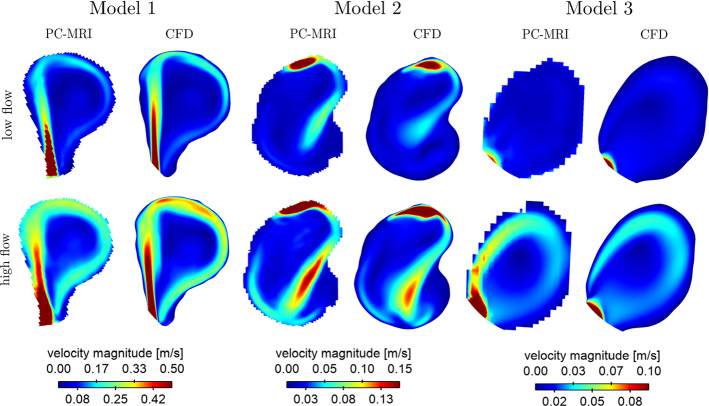
Qualitative illustration of time-averaged intra-aneurysmal velocity based on PC-MRI measurements and CFD simulations. Top: Results acquired with the low flow measurement set-up. Bottom: Results acquired with the high flow measurement set-up

Overall, Fig. 3 shows a qualitatively good agreement in the flow behaviour between PC-MRI measurements and CFD simulations. In accordance with the VW-MRI results (Fig. 2), the general flow characteristics are similar within one model for low and high flow conditions. As indicated from the VW-MRI measurement, the intra-aneurysmal flow differs in magnitude and complexity among the models M1 – 3. The flow entering the aneurysm was different for each model due to varying flow rates, numbers of side branches and parent vessel structures. As calculated based on haemodynamic simulations, the aneurysm in model M1 is fed with the highest flow (low flow average 158 ml/min; high flow average 268 ml/min). This correlates with the strong suppression of blood flow observed in Fig. 2. Moreover, the inhomogeneous enhancement in the aneurysm sac and near the aneurysm neck can be found in the regions, where the lowest intra-aneurysmal flow is observed. Model M2 experiences an average flow rate entering the aneurysm of 56 ml/min for low flow conditions and 77 ml/min for high flow conditions as calculated based on haemodynamic simulations. The inflow jet position correlates with the lower enhanced regions for low and high flow conditions. Nevertheless, the origin of the signal void zones between enhanced regions remains unclear and requests a more detailed investigation of the intra-aneurysmal flow behaviour.

In accordance with the high enhancement seen for model M3 in the VW-MRI (recall Fig. 2), only a small fraction of the parental flow enters the aneurysm (0.1 and 0.2 ml/min). Nevertheless, the lower enhancement observed under the high flow conditions correlates with the position of the aneurysm jet (Fig. 3).

**Fig. 4:**
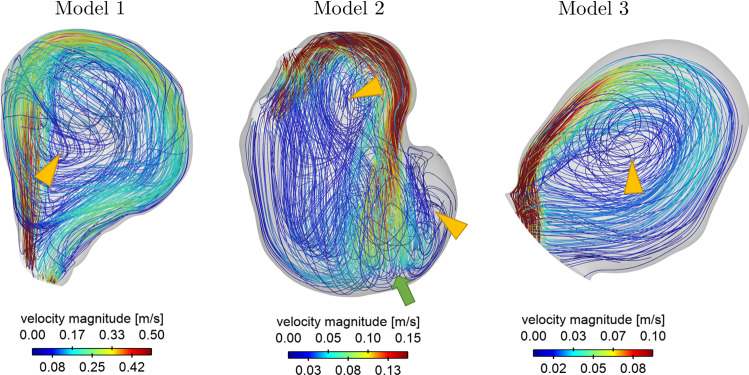
Streamline illustration of CFD simulations for high flow inlet conditions. Recirculation zones are marked with orange triangles, the impingement zone is represented with a green arrow

The streamlines illustrated in Fig. 4 indicate the flow complexity inside the aneurysmal lumens. Models M1 and M3 possess one recirculation zone (orange arrow), whereas the flow in model M2 seems to have a higher complexity. Here, flow division is observed at the impingement zone in the aneurysm sac (green arrow) and two recirculation zones (orange triangles) can be observed.

**Fig. 5:**
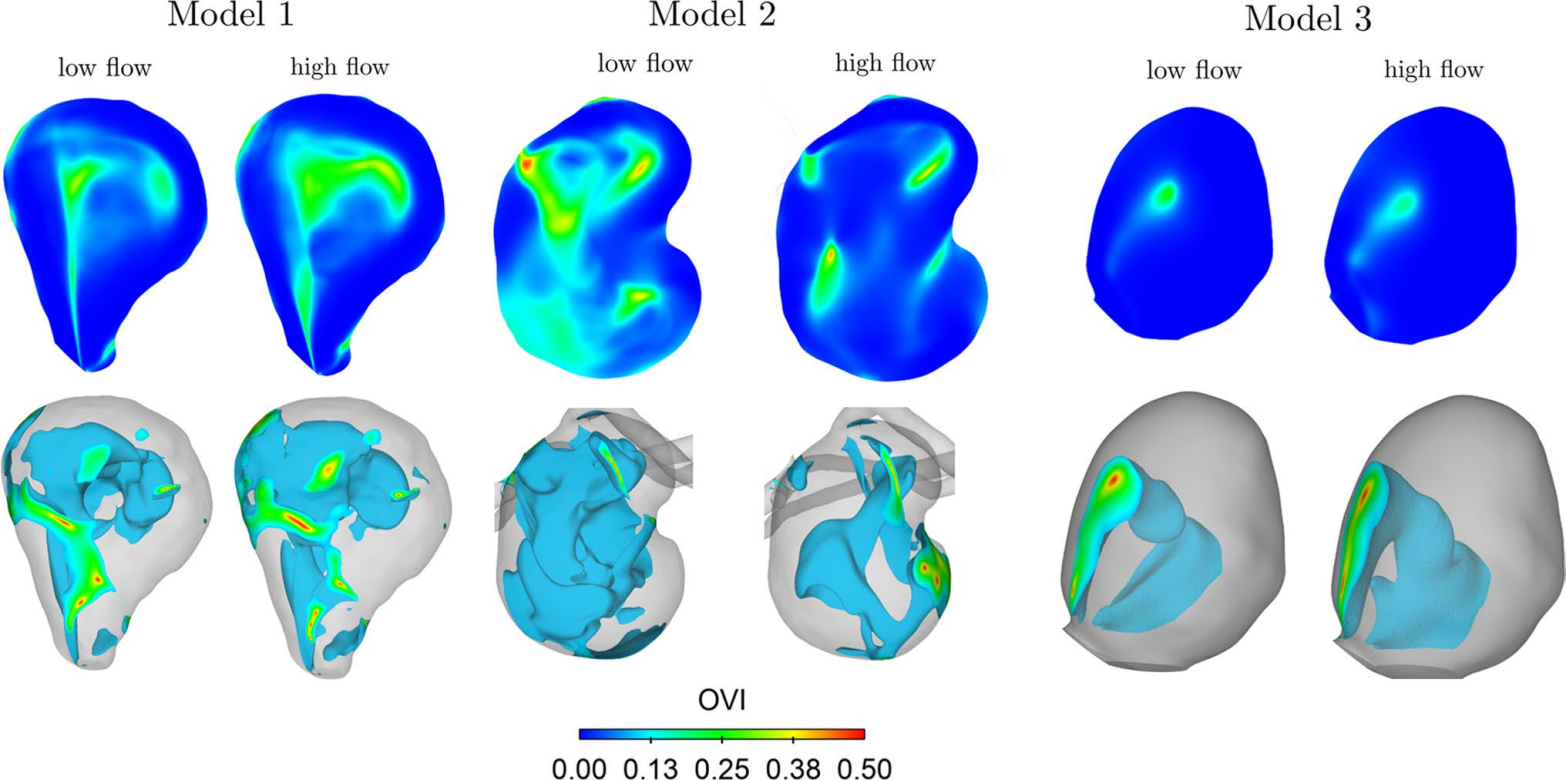
Illustration of the flow complexity over the cardiac cycle. Top: OVI centre slice distribution. Bottom: Isovolumes representing regions with an OVI above 0.1

The OVI indicates the flow complexity throughout the cardiac cycle (Fig. 5). All aneurysm models show the highest temporal variation in the centre of the lumen. The isosurfaces represent an OVI value above 0.1. The OVI distribution is more heterogeneous for model M1 and M2 (low flow conditions), but confined for model M3 and M2 (high flow conditions). No direct colocalization between signal suppression zones (Fig. 1) and high temporal complexity can be observed.

### Quantitative relation between intra-aneurysmal velocity and signal intensity

**Fig. 6:**
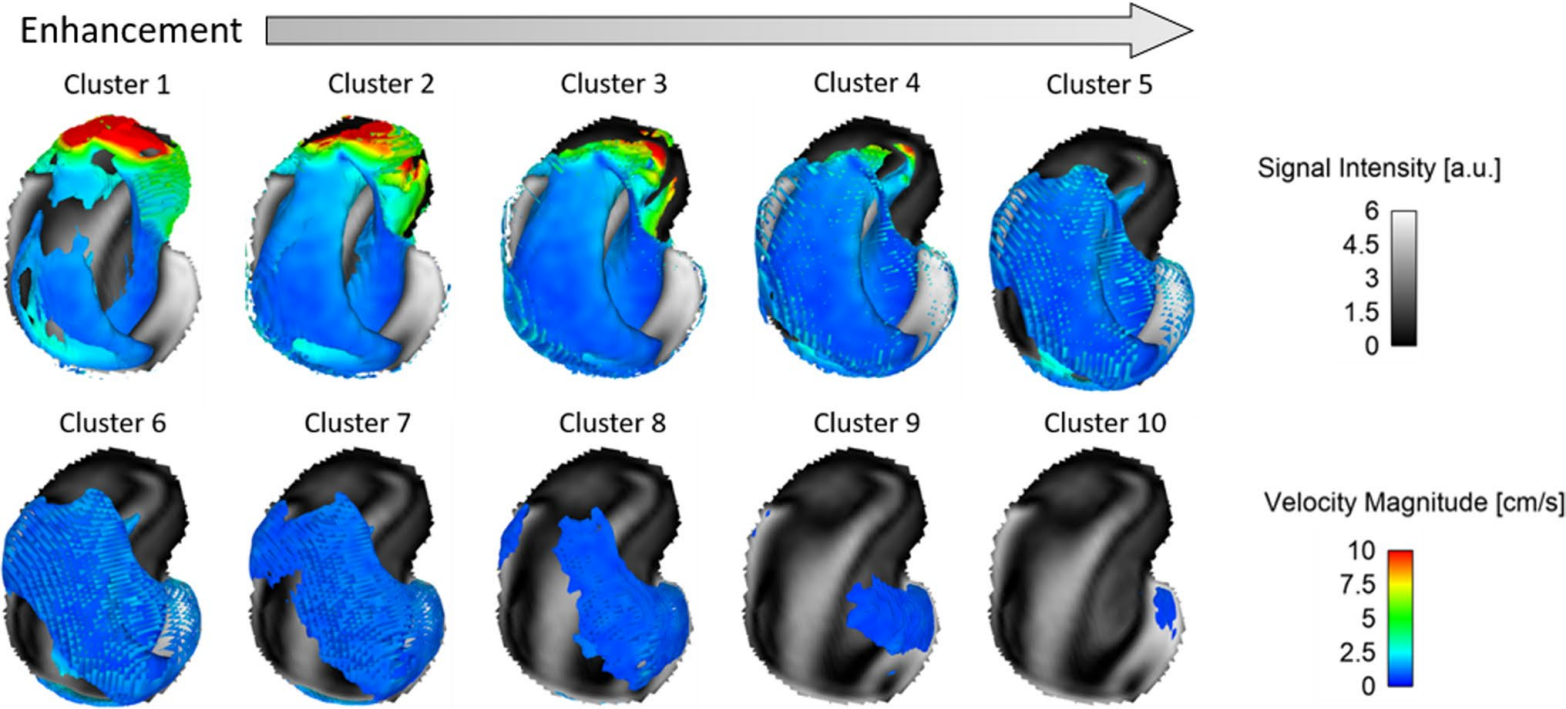
Signal intensity clusters colour-coded with velocity magnitude from PC-MRI measurements for model M2. The signal values were separated into ten equivalent regions, cluster 1 correlates with the lowest signal intensity values and cluster 10 with the highest. The number of voxel values within one cluster changes as the signal values are not evenly distributed over the whole range

To assess the relationship between signal intensity in the VW images and the measured velocity, signal and velocity values in ten different clusters (see 2.4) are evaluated. Figure 6 indicates the velocity distribution within the clusters for the representative model M2. Low flow can be observed in all clusters, thus in all signal regimes, whereas high flow can only be found in regions correlating with low signal. Therefore, low signal intensity areas do not necessarily correspond to high flow and low flow does not necessarily correspond to a high signal. The effect gets smaller with increasing signal intensity or higher flow, respectively. Similar observations can be found for the other models and flow regimes.

**Fig. 7:**
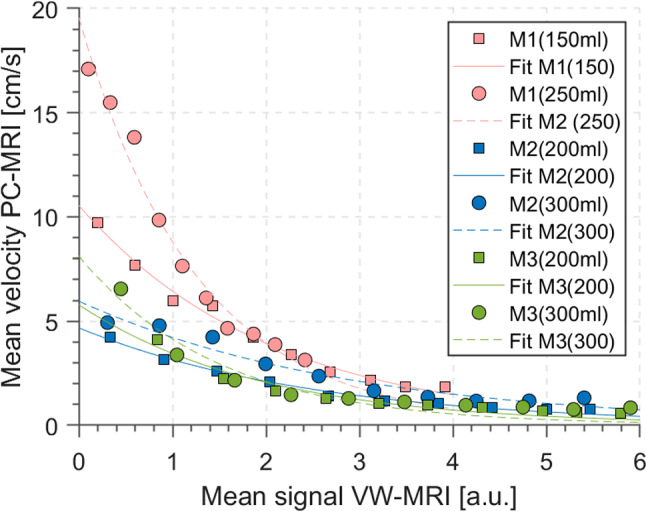
Mean velocity measured using PC-MRI over mean signal intensity measured for all models within the generated clusters. Exponential fits have been generated for each model

Figure 7 illustrates mean velocity measured using PC-MRI plotted over mean signal intensity measured for all models within the generated clusters. For each model, a similar dependence between velocity and signal enhancement can be found. Clusters with lower average signal intensities are associated with higher average velocity values. In the signal range between three and six, the average velocity for all models is in the same range (3–0 cm/s) and linearly decreases, although for model M1 the enhancement is never above four. In the signal range between zero and three, the average velocity decreases exponentially. Nevertheless, this exponential value is highly different among the models.

## Discussion

This study quantifies the relationship between luminal signal intensity based on VWI and the velocity distribution inside intracranial aneurysms. Three representative patient-specific IAs that differ in size and shape, as well as in intraluminal flow magnitude, have been evaluated. Qualitatively, different enhancement patterns as well as flow fields have been observed. The highest signal intensity and lowest inflow rate occur in model M3, while the lowest enhancement and largest inflow rate were found in model M1. The observed flow phenomena, detected in good agreement using PC-MRI or CFD, match with VW signal intensity. Inflow jets and low flow regions colocalize with low and high intensity zones. Furthermore, complex enhancement patterns, including signal void zones in between enhanced regions, indicate a higher complexity of the intra-aneurysmal flow (model M2). These findings support the hypothesis that behaviour and characteristics of the intra-aneurysmal flow highly influence the flow suppression and consequently signal intensity observed by the VWI measurements.

The qualitative results are supported by a quantitative evaluation of the relationship between signal intensity and measured velocity values. Although the overall trend indicates that higher signal intensity is generally associated with lower intra-aneurysmal flow, low signal intensity can also be found in regions corresponding to all other flow regimes. More specifically, luminal flow enhancement can be almost exclusively found for slow flow areas, but the effect is not very distinct in signal-suppressed regions.

The nature of the blood flow within IAs can be complex and highly patient-specific. Thus, phenomena contributing to flow suppression can occur in parallel. Based on the results of the associated study, it is assumed that the suppression can not only be attributed to high flow, but further factors associated with complex spatial behaviour have an additional impact. Nevertheless, no direct conclusion can be drawn from the temporal complexity (e.g., based on the evaluation of OVI) to regions that face a signal suppression during VW-MRI.

Previous studies have discussed the role of signal intensity and argued about fact or artefact [3, 19, 25, 26, 35]. This study encourages using the enhancement patterns as a feature and potential biomarker to identify pathological changes in the luminal flow behaviour. Due to the high complexity of the aneurysmal flow, VWI cannot completely replace more sophisticated measurement methods like PC-MRI on a quantitative basis. Nevertheless, it can give an estimate of the overall flow range and magnitude measured. Moreover, the enhancement patterns can be used for the characterization of flow phenomena and comparison of flow in patients with multiple aneurysms. Advantages of VW-MRI in comparison to PC-MRI are the smaller measurement times and reduced number of associated post-processing steps. Nevertheless, the potential relationship between luminal enhancement and IA rupture requires further in-depth investigation. However, this study contributes to an improved understanding of previous observations and occurring flow-related phenomena.

Apart from the findings, it is important to emphasize that this study has several limitations: Due to the complex manufacturing and measurement process, the number of models was limited to three. Furthermore, the high difference in size and shape of the models has ensured the investigation of a broad range of flow regimes. Nevertheless, this leads to a lack of information about the behaviour in aneurysms covering a similar flow behaviour. Additionally, the use of a blood-mimicking fluid could lead to different signal intensities in comparison to real blood measurement. Finally, no information about wall enhancement could be given because of the *in vitro* character of the study.

Future studies will include the VW-MRI results within similar aneurysm types to ensure comparability and representativeness of the results, including *in vivo* investigations. Furthermore, a strong focus will set on the detailed identification and characterization of additional factors that contribute to flow suppression or signal enhancement.

## Conclusions

This study has demonstrated the influence of intra-aneurysmal flow behaviour on VW-MRI signal intensity. It supports the use of intra-aneurysmal flow-related signal enhancement as a feature to characterize the overall flow behaviour. VW-MRI can help to qualitatively assess flow phenomena, including inflow jets, recirculation zones and different flow regimes. It is well suited to compare the flow regimes in different aneurysms. On a qualitative basis, VW-MRI can be an alternative to complex PC-MRI measurements or support spatially lower-resolved measurements in detail and information. The investigation of signal intensity patterns can help to identify pathological changes in the aneurysmal lumen.

## Declarations

### Availability of data and material

Processed data and code is available upon request from the corresponding author.
